# Pamphlets Received

**Published:** 1876-06

**Authors:** 


Pamphlets Received.—Remarks on Urethral Stricture. By Fessenden N. Otis, M.D., Professor of Genito-Urinary Diseases, College of Physicians and Surgeons, New York.

On Stricture as the Initial Cause of Gleet, with remarks on Urethral Calibre. Being a reply to the paper of Dr. H. B. Sands on the same subject. By Fessenden N. Otis, M.D., Professor of Genito-Urinary Diseases, College of Physicians and Surgeons, New York, Surgeon to Charity Hospital, etc.

A series of American Clinical Lectures. Edited by E. C. Seguin, M.D.: Some forms of Dyspepsia. By Francis Delafield, M.D., Adjunct Professor of Pathology and Practical Medicine in the College of Physicians and Surgeons, New York. Certain forms of Morbid Sensibility. By J. S. Jewell, M.D., Professor of Nervous and Mental Diseases in Chicago Medical College.

The Treatment of Mild Cases of Melancholia at Home. By E. C. Seguin, M.D., Clinical Professor of Diseases of the Mind and Nervous System, College of Physicians and Surgeons, New York.

Neuralgia and other Neuroses arising from Cicatrices of the Scalp, and their Surgical Treatment. By Frederick D. Lente, M.D.

Medico-Legal Evidence of Independent Life in a New-born Child. By J. B. Gaston, M.D., of Montgomery, Ala.

